# Bacterial lipids: powerful modifiers of the innate immune response

**DOI:** 10.12688/f1000research.11388.1

**Published:** 2017-08-07

**Authors:** Courtney E. Chandler, Robert K. Ernst

**Affiliations:** 1 Department of Microbial Pathogenesis, University of Maryland, Baltimore, 650 W. Baltimore Street, 8th Floor South, Baltimore, MD, 21201, USA

**Keywords:** lipoproteins, LPS, LTA, TLR2, TLR4, innate immunity

## Abstract

The innate immune system serves as a first line of defense against microbial pathogens. The host innate immune response can be triggered by recognition of conserved non-self-microbial signature molecules by specific host receptor proteins called Toll-like receptors. For bacteria, many of these molecular triggers reside on or are embedded in the bacterial membrane, the interface exposed to the host environment. Lipids are the most abundant component of membranes, and bacteria possess a unique set of lipids that can initiate or modify the host innate immune response. Bacterial lipoproteins, peptidoglycan, and outer membrane molecules lipoteichoic acid and lipopolysaccharide are key modulators of the host immune system. This review article will highlight some of the research emerging at the crossroads of bacterial membranes and innate immunity.

## Introduction

The innate immune system serves as a first barrier protecting the host from microbial pathogens, including bacteria, viruses, parasites, and fungi. At the most basic level, the innate immune system is composed of a set of cells and molecules aimed at recognizing and responding to a wide range of microbial stimuli. Specifically, a series of germline-encoded receptors and secreted proteins have evolved to recognize common, conserved, and indispensable microbial features called pathogen-associated molecular patterns (PAMPs) ^1, 2^. PAMPs are molecules shared by and exclusive to microbes and are recognized by host immune cell receptors called pattern recognition receptors (PRRs). PRRs may be present on the external cell membrane or internally in endosomes of host innate immune cells such as leukocytes, macrophages, dendritic cells, and natural killer cells ^1^.

**Figure 1.**
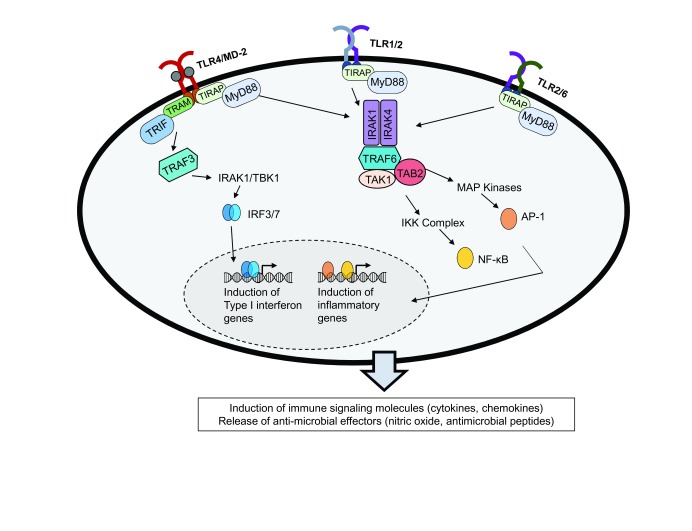
Toll-like receptor (TLR) stimulation, signaling, and immunological outcomes. Microbial pathogen-associated molecular patterns (PAMPs) recognized by TLRs lead to initiation of signaling cascades in a MyD88-dependent manner. TLR4 may also signal through an alternative TRIF/TRAM pathway. The signals from both pathways converge on specific transcription factors, which translocate to the nucleus and upregulate immune genes involved in responding to microbial stimuli. The canonical outcomes of TLR4 and TLR2 signaling are upregulation of inflammatory cytokines, which in turn can help recruit other immune cells and promote anti-microbial effectors.

The Toll-like receptors (TLRs) are the best characterized of the PRRs. Currently, 10 TLRs in humans and 13 in mice have been described, and each recognizes unique microbial PAMPs ^1, 2^ ( Table 1). Upon recognition of bacteria-specific extracellular features, specific combinations of TLRs will dimerize. Bacterial lipoproteins, lipoteichoic acid (LTA), and peptidoglycan (PGN), which signal through TLRs 1, 2, and 6, and lipopolysaccharide (LPS), which signals through TLR4, will be the focus of this mini-review ^3, 4^. TLRs can function as homo- or hetero-dimers; the exception is TLR5, which can also function as a monomer. TLR4 forms a homo-dimer, whereas TLRs 1, 2, and 6 can form hetero-dimers, thereby changing their PAMP specificity. TLR2 can dimerize with TLR1 to recognize bacterial lipoproteins and parasitic surface molecules. When paired with TLR6, this hetero-dimer can recognize a more diverse group of microbial surface molecules such as PGN, yeast, and lipoproteins ^1– 3^. Upon binding of a microbial ligand, TLR molecules initiate a signaling cascade that converges on a group of molecular effectors, including pro-inflammatory transcription factors, which result in upregulation of genes and processes involved in the host immune response ( Figure 1). TLR signaling cascades ultimately result in the production of chemokines, cytokines, and host anti-microbial molecules ^1, 2^. TLR1/2, TLR2/6, and TLR4 use two specific cytosolic adaptor proteins—MyD88 and TIRAP—to initiate intracellular signaling cascades upon stimulation. MyD88 recruits IRAK-4, which in turn activates downstream proteins, including transcription factor NF-κB and MAPKs JNK and p38 MAPK. Activated transcription factors translocate to the nucleus and initiate upregulation of immune effector genes. TLR4 can also signal through the alternative TRIF/TRAM pathway, leading to induction of different immunological mediators (reviewed by De Nardo ^2^ in 2015).

**Table 1.** Toll-like receptors and their associated pathogen-associated molecular patterns.

Toll-like receptor	Pathogen-associated molecular pattern recognized
TLR3, TLR7, TLR8, and TLR9	Nucleic acid
TLR1/2	Lipoproteins, lipoglycans, parasitic surface molecules, and peptidoglycan
TLR2/6	Lipoproteins
TLR4	Lipopolysaccharide
TLR5	Flagellin
TLR10	Parasitic proteins

The Toll-like receptors (TLRs) are pattern recognition receptors that recognize specific microbial features. In humans, 10 TLRs have evolved to recognize a variety of microbial molecules. TLR1/2, TLR2/6, and TLR4 are crucial for recognition of bacterial lipid molecules.

Microbes contain a number of characteristic lipids and lipoproteins. These molecules are crucial not only for membrane integrity of the bacteria but also for signaling, responding to environmental stresses, and propagation. The characteristics, structure, and specific mechanisms leading to immune activation of these bacterial lipids represent long-standing research questions of interest, largely due to the immunological consequences of such activation. Better understanding of innate immune activation could improve therapeutic approaches to bacterial infections. Furthermore, this knowledge could help prevent out-of-control immunological signaling, as is present in conditions such as sepsis. This mini-review aims to describe the most recent and emerging research in this field.

## The TLR2 agonist: an unclear picture

**Figure 2.**
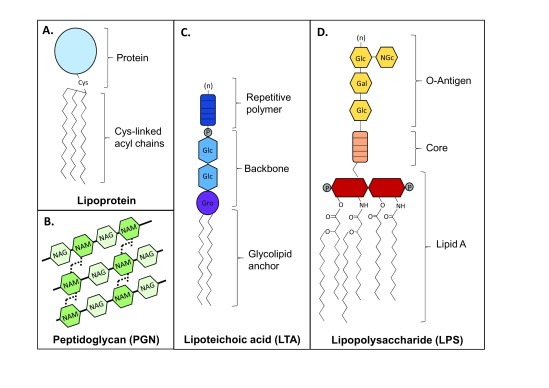
Structural variety of microbial lipid pathogen-associated molecular patterns. ( **A**) Lipoproteins are anchored to the bacterial membrane by cysteine-linked acyl chains and have been described in both Gram-positive and Gram-negative bacteria. ( **B**) Peptidoglycan (PGN) consists of a sugar polymer composed of β-(1,4)-linked *N-*acetylglucosamine (NAG) and *N-*acetyl muramic acid (NAM). Peptide chains of three to five amino acids are attached to the NAM residues and cross-linked to a chain from another sugar polymer strand, creating a mesh-like structure. ( **C**) Lipoteichoic acid (LTA) is a Gram-positive surface polymer abundant in the bacterial envelope. The LTA structure can vary and is classified into categories by characteristics such as its backbone type and repetitive polymer composition (types I–V). Many LTAs contain a glycerophosphate (GroP) backbone structure attached to a glycolipid anchor. The repeating unit (marked with “n”) can also vary and may contain glycerol, glycose, galactose, ribitol, phosphate, and other related derivatives. Type I/II LTA backbone is represented here. ( **D**) Lipopolysaccharide (LPS) is a major component of the outer leaflet of the outer membrane of Gram-negative bacteria. The hydrophobic membrane anchor, lipid A, can vary in acyl chain length and number and can be mono- or bis-phosphorylated. The lipid A molecule is responsible for innate immune recognition by TLR4. Lipid A is connected to a hydrophilic core polysaccharide chain, followed by a repeating (marked with “n”) oligosaccharide chain (the O-antigen), which is specific to the bacterial serotype.

**Figure 3.**
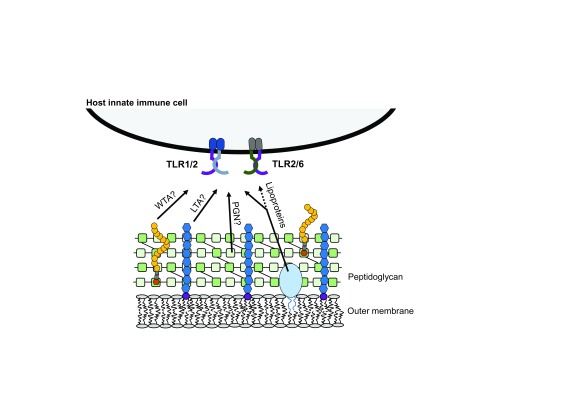
Key stimulatory molecules from Gram-positive bacteria. Lipoteichoic acid (LTA), wall teichoic acid (WTA), peptidoglycan (PGN), and tri-acylated lipoproteins are constituents of the Gram-positive bacterial cell wall and have been proposed to signal through TLR1/2 and TLR2/6. TLR, Toll-like receptor.

Gram-positive bacteria possess a unique set of components that are involved in pathogenesis, including lipoproteins, PGN, poly- *N*-acetyl glucosamine, wall teichoic acid, and LTA ^5^. These molecules are structurally unique. Lipoproteins are proteins anchored to the membrane by cysteine-linked fatty acids and are present in both Gram-positive and Gram-negative bacteria ^6, 7^ ( Figure 2A). Typically, lipoproteins are tri-acylated in Gram-negative bacteria and di-acylated in Gram-positive bacteria and Mycobacterium ^8, 9^. The canonical triacyl form is proposed to be recognized by TLR1/2, and the diacyl form by TLR2/6 ^10^. PGN is a polymer of *N*-acetylglucoasmine and *N*-acetylmuramic acid sugars and amino acids, forms a thick mesh outside the membrane of Gram-positive bacteria, and is present between the dual membranes of Gram-negative bacteria ^11^ ( Figure 2B). LTA is an amphiphilic molecule specific to Gram-positive bacteria and commonly contains a glycerophosphate backbone and glycolipid membrane anchor ^12^ ( Figure 2C). Host immune recognition of these molecules has been shown to require TLR2 (paired with either TLR1 or TLR6) for activation ( Figure 3). Historically, it was thought that LTA and PGN were the primary stimulators of TLR2 upon bacterial colonization and infection. However, as targeted knockout bacteria mutant strains and improved purification procedures for the different ligands are developed, a new dogma has emerged that suggests lipoproteins may be the main activators of TLR2 ^13, 14^.

Recent studies using lipoprotein- or LTA-specific mutations and purified ligand preparations support the idea that lipoproteins are the dominant trigger of TLR2 signaling. Kim *et al.* concluded that *Streptococcus gordonni* lipoproteins were required for the induction of nitric oxide (NO) in a TLR2-dependent manner from mouse macrophages, as lipoprotein mutants were unable to induce NO in contrast to specific LTA mutants and heat-killed cells ^15^. Studies using *Staphylococcus aureus* showed that lipoproteins are essential for interleukin-8 (IL-8) expression in human intestinal epithelial cells and LTA alone is unable to enhance dendritic cell macropinocytosis, as compared with PGN ^16, 17^. Furthermore, using mutant strains of *Enterococcus faecalis* that showed a threefold higher lipoprotein content, Theilacker *et al*. observed that these strains were more potent activators of macrophages and that this potency was lost upon inactivation of lipoprotein biosynthesis ^18^. The authors additionally showed that these strain types led to increased induction of key cytokines, such as tumor necrosis factor-alpha, IL-6, and macrophage inflammatory protein 2, and increased influx of leukocytes and mortality in mice. Martinez de Tejada *et al*. extended these studies to show that lipoproteins are the most potent stimulators of TLR2 *in vitro* and are capable of producing sepsis-like pathology in mice ^19^. Finally, evidence even suggests that LTA can serve to dampen lipoprotein-induced immune cell stimulation and IL-8 production in intestinal epithelial cells ^20^. This research collectively reinforces the role of bacterial lipoproteins as immune stimulators.

Despite emerging evidence of lipoprotein-dependent TLR2 selectivity, studies still support the classic view of LTA as an agonist of TLR2. Purified LTAs from *Weissella cibaria* and *Bacillus subtilis* were used to study the downstream immune effects after cell exposure. These studies describe an upregulation in TLR2-associated cytokine production and inflammatory markers, supporting the idea of LTA as a significant innate immune stimulatory molecule ** ^21, 22^. Furthermore, Wang *et al*. showed that LTA from *Clostridium butyricum* inhibited inflammatory response and apoptosis induced by *S. aureus* LTA in cells, suggesting a specific structure-activity relationship between LTA and TLR2 activation ^23^.

The ability to identify PAMP structural characteristics specifically responsible for the initiation and perturbation of the innate immune response is the topic of much research. Insight into why and how particular features activate PRRs would provide a better understanding of innate immunity initiation and could highlight potential points for therapeutic intervention. Reports of crystal structures of TLR2/1 and TLR2/6 in complex with their ligands have allowed molecular-level detail regarding the receptor-ligand interaction ^24, 25^. With the goal to further define the minimal structural scaffolding required for TLR2 activation, Jiménez-Dalmaroni *et al*. recombinantly expressed the human TLR2 ectodomain and analyzed its ligand-binding properties. They showed that the diacylglycerol component of microbial glycolipids and lipoproteins is the ligand that binds to the ectodomain of hTLR2. However, they did not complex TLR2 with either TLR1 or TLR6 or study it in a whole-cell environment ^26^.

The current research supports both lipoproteins and LTA as TLR2 signaling molecules, yet the ‘dominant’ TLR2 trigger remains an active area of study. The dual-associative nature of TLR2 when complexed with TLR1 or TLR6 compounds the issue of identifying a single dominant molecular trigger, although continued research will yield valuable insight into the roles of specific subclasses of membrane components in pathogenesis.

## Gram-negative lipoproteins and their rising roles in immune stimulation

Gram-negative bacterial lipoproteins have been shown to be involved in colonization, invasion, evasion of host immune defenses, and immunomodulation during infection ^7, 27^. It was generally believed that lipoproteins in Gram-negative bacteria reside in either the inner membrane or inner leaflet of the outer membrane. Recent research suggests that surface-exposed lipoproteins are more common than previously thought among Gram-negative bacteria ^27^. Based on specific search algorithms, most bacteria are predicted to encode about 75 to 200 lipoproteins, many of which have yet to be characterized ^27^. As our understanding of bacterial lipoproteins has evolved, it has prompted study of a number of less well-understood Gram-negative bacterial pathogens. Wang *et al.* used recombinantly expressed lipoproteins (predicted) from the obligate intracellular pathogen *Chlamydia trachomatis* to test for their ability to stimulate mouse and human macrophage cell lines. In addition to identifying stimulatory lipoproteins, they identified that the signaling pathways initiated after induction were mediated through TLR1/2 and TLR2/CD14 ^28^. Dennehy *et al*. described a specific PGN-associated lipoprotein important for host cell attachment and cytokine secretion in *Burkholderia cepacia* complex, which commonly colonizes the airways of patients with cystic fibrosis ^29^. *Fusobacterium nucleatum*, an oral cavity commensal, possesses a characteristic cell wall–associated protein that has been shown to induce specific anti-microbial immunity by activating both TLR1/2 and TLR2/6 ^30^. Despite increased interest in recent years, the field is just beginning to understand the multitude of ways in which Gram-negative lipoproteins play a role in innate immune initiation, evasion, and bacterial pathogenesis.

## Lipopolysaccharide, the major immune mediator of Gram-negative bacteria

**Figure 4.**
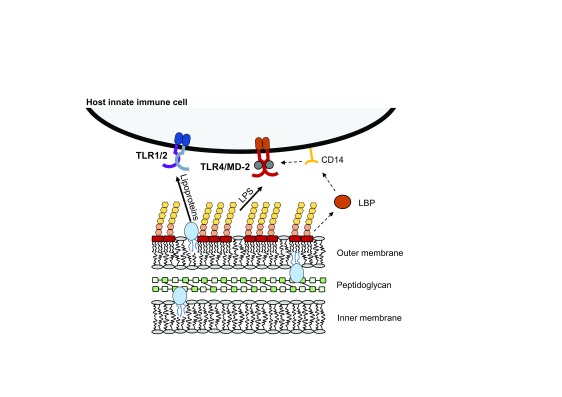
Key stimulatory molecules from Gram-negative bacteria. The outer leaflet of the outer membrane of Gram-negative bacteria is largely composed of lipopolysaccharide (LPS) with intermittent lipoproteins and phospholipids. The membrane anchor lipid A portion of LPS is the key stimulatory molecule for TLR4 on the host cell membrane. Lipid A can be extracted from the bacterial membrane by LPS-binding protein (LBP), which transfers it to membrane-bound CD14. CD14 transfers lipid A to MD-2 within the TLR4 pocket, leading to intracellular stimulation. Tri-acylated Gram-negative lipoproteins are thought to be recognized by TLR1/2. TLR, Toll-like receptor.

Gram-negative bacteria possess a characteristic lipoglycan molecule that composes the majority of the outer leaflet of their outer membrane. This molecule is called LPS, or endotoxin, and is composed of three distinct regions: a repetitive glycan polymer, oligosaccharide core, and membrane-anchor lipid A molecule ^31^ ( Figure 2D). Lipid A is the ligand for the TLR4 complex. TLR4 acts in concert with the accessory protein MD-2 to initiate an immune response with MD-2 necessary for the physiological recognition of lipid A. Upstream of TLR4/MD-2, a soluble protein called LPS-binding protein (LBP) extracts lipid A from lipid micelles or the bacterial membrane and transfers it to the glycosylphosphatidylinositol-linked surface protein CD14, which in turn transfers lipid A to the TLR4/MD-2 complex ^4, 31^ ( Figure 4). The structural mechanism of lipid A binding was initially described by Park *et al*. following co-crystallization of different lipid A structures with components of the TLR4/MD-2 complex ^32, 33^. Recently, Kim and Kim further revealed the dynamic transfer cascade of LPS from LBP and CD14 to the TLR4/MD-2 complex ^34^. They identified that the interaction between LBP and CD14 is rapid and transient, on the millisecond time scale, and is mediated by electrostatic interactions between a basic amino acid patch on LBP and an acidic patch on CD14. After binding one molecule of LPS, CD14 rapidly dissociates from the LBP/LPS complex, mediated by electrostatic repulsion. CD14 then delivers a single LPS molecule to MD-2 within the TLR4 pocket and in a TLR4-dependent manner ^34^. This work provided the finest level of detail to date about the binding and transfer of LPS to TLR4.

The initiation and intensity of the host immune response can be dependent on lipid A structure. Specifically, lipid A structural changes in regard to the terminal phosphate groups and attached acyl groups can alter immunological outcomes through altered TLR4 complex specificity ^35, 36^. Recent studies have further highlighted how closely linked lipid A structural modification is to immunological initiation and outcome. Studies of patients infected with *Neisseria meningitidis* have revealed that variable lipid A structure can have multifaceted effects on immune responses which in turn have a critical impact on the nature of meningococcal infection. Hellum *et al*. observed that patients infected with *N. meningitidis* with penta-acylated lipid A, as compared with wild-type hepta-acylated lipid A, had less systemic inflammation and coagulopathy and that this was correlated with a weaker induction of monocytes ^37^. John *et al*. observed that lipid A in clinical isolates of *N. meningitidis* had lower levels of terminal phosphorylation and that this correlated with septicemia ^38^. They also observed that lipid A from all invasive strains was hexa-acylated but that carrier strains from colonized patients were penta-acylated, supporting the previous data by Hellum *et al*.

Using *in vitro* transcriptome analysis, Luan *et al*. expanded on the idea that LPS structural changes alter immunological outcome ^39^. They investigated human leukocyte responses to stimulation with pro-inflammatory canonical LPS and compared them with those from stimulation with less-inflammatory mono-phosphorylated lipid A. Although both molecules induced transcriptome profiles that were largely similar, the canonical LPS structure was a more potent inducer of pro-inflammatory cytokine transcripts, further supporting the idea that structural modifications of LPS can be used to alter immunological outcome ^39^. Malgorzata-Miller *et al*. highlighted the potential benefits of LPS-controlled immunomodulation by using a novel lipooligosaccharide (LOS) from *Bartonella quintana*, the causative agent of trench fever ^40^. The lipid A structure of this microbe is unusual in that it contains an unsaturated acyl chain and a long 26-carbon fatty acid. When isolated, this LOS performed as a TLR4 antagonist and anti-inflammatory mediator in the presence of highly inflammatory *E. coli* LPS ^40^. This work adds to our understanding of the structural features associated with lipid A antagonism, which have been previously described ^41, 42^. Collectively, these studies highlight how important even subtle LPS structural changes are to immunological outcome, including TLR4 activation, the inflammatory response, and disease progression. Further work needs to be done to better define the downstream outcome of specific LPS modifications. A better understanding of the structure-activity relationship will allow for better therapeutic targeting to prevent immune dysregulation-related patient outcomes, such as sepsis.

## Membrane vesicles: an emerging field

**Figure 5.**
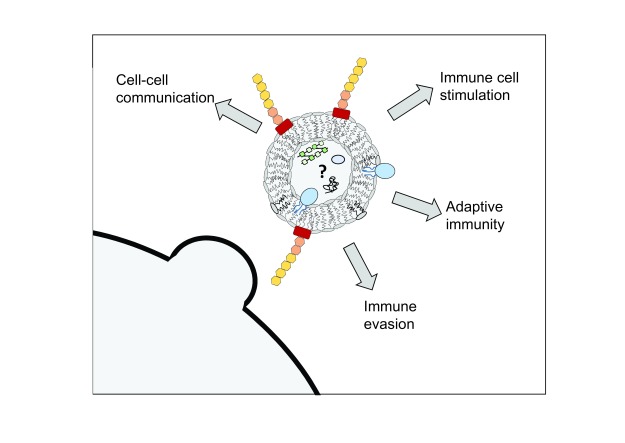
Immunological outcomes mediated by membrane vesicles (MVs). MVs can be generated from both Gram-positive and Gram-negative bacteria (representative Gram-negative MV shown here). MVs are proposed to contain many of the membrane-associated bacterial molecules with cargo that may contain both membrane- and cytosolic-derived proteins, lipids, and nucleic acid. MVs have been described to be involved with cell-cell communication (between both bacterial and host cells), immune cell stimulation, long-term adaptive immunity, and immune evasion mechanisms.

Membrane vesicles (MVs) represent an active and evolving area of research. Gram-negative MVs (commonly called outer membrane vesicles) and Gram-positive MVs (commonly called extracellular vesicles) were initially thought to be a byproduct of bacterial growth. However, it is now widely accepted that MVs are produced by both Gram-negative and Gram-positive bacteria and can have roles in antigen delivery, cell-cell communication, immune responsiveness, and inflammation ^43, 44^ ( Figure 5). MVs are composed of an array of molecules derived from the bacterium and can include PGN, LPS or LTA, DNA, RNA, enzymes, and proteins from membrane, periplasm, and cytoplasm. The exact molecular cargo and size of the vesicle can vary between bacterial species and environments, and cargo contents remain an active area of study ^45^. Despite the many recent advances in MV research, the mechanisms underlying their biogenesis remain poorly understood for both Gram-negative and Gram-positive bacteria. It is thought that Gram-negative MVs are generated in a ‘pinching-off’ type of mechanism. Gram-positive bacteria are assumed to generate MVs by using a different mechanism, as it is thought that a pinching-off mechanism is hindered by the large extracellular PGN layer in the bacterial membrane ^43, 44^.

Improved understanding of bacterial membranes and isolation techniques have led to a vast increase in information detailing the biological effects of both Gram-negative and Gram-positive MVs. Recent work by Jurkoshek *et al*. suggests that bacterial MVs can serve as a means for interspecies communication between pathogenic bacteria and host immune cells ^46^. Vanaja *et al.* recently showed that internalization of Gram-negative MVs by host immune cells is responsible for cytosolic localization of LPS, which triggers specific immune responses, such as inflammasome activation ^47^. It has also been recently shown that bacterial membrane composition can influence MV production. Elhenawy *et al*. showed that deacylation of LPS altered the level of MV production in *Salmonella enterica* serovar Typhimurium and that LPS deacylation may inversely correlate with other LPS modifications, suggesting a synergy of host evasion strategies ^48^. The downstream immune effectors resulting from MV exposure provide further insight into the roles of MVs in immunity and infection. MVs isolated from species such as *Legionella pneumophila*, *Clostridium difficile*, *Porphyromonas gingivalis*, *N. meningitidis*, and *S. aureus* have displayed a range of downstream immunological outcomes, including those mediated by TLR2 and TLR4 ^49– 53^.

MVs contain not only bacterial lipids but also membrane proteins, receptors, and other molecules ^43, 44^. Owing to their antigenic variability and small size, MVs are actively being investigated as vaccine candidates, which has become an entire research field of its own. MVs from several bacterial species, including *Bordetella pertussis*, *Streptococcus pneumoniae*, *N. meningitidis*, *S. aureus*, *Salmonella*, and *Klebsiella pneumoniae*, have shown favorable immunological outcomes after vaccination studies ^54– 59^. The recent work on MVs is vast, and several review articles have highlighted the developing research on bacterial MVs, including the broadly encompassing article by Pathirana and Kaparakis-Liaskos ^43^ from 2016.

## Conclusions

Bacterial membranes have long been sources of study, especially in regard to immune stimulation and evasion. Recent research highlights how complex the interaction between the microbe membrane and host immunity can be. Even well-studied phenomena, such as TLR4 activation by LPS, still have many unknowns that warrant further research. The many structural variations of lipid A and LPS and their downstream effects on innate immune initiation and response will continue to be a question of interest despite recent research helping to better illuminate some structure-activity relationships in this arena. Research on lipoproteins and their roles in pathogenicity may help illuminate alternative targets for bacterial control and eradication. Additionally, this line of research will help better define overlooked players in pathogenicity. Perhaps the most rapidly expanding field of research is that of bacterial membranes, from both Gram-negative and Gram-positive bacteria. This research will help better define membrane processes critical to bacterial survival, inter-cell communication mechanisms, and immune evasion and modulation and may provide viable vaccine options for hard-to-treat bacteria. The bacterial membrane is complex, and only by addressing all of the players will we be able to better understand the host-microbe interaction.

## Abbreviations

IL, interleukin; LBP, lipopolysaccharide-binding protein; LOS, lipooligosaccharide; LPS, lipopolysaccharide; LTA, lipoteichoic acid; MV, membrane vesicle; NO, nitric oxide; PAMP, pathogen-associated molecular pattern; PGN, peptidoglycan; PRR, pattern recognition receptor; TLR, Toll-like receptor.
